# Extracting Objects for Aerial Manipulation on UAVs Using Low Cost Stereo Sensors

**DOI:** 10.3390/s16050700

**Published:** 2016-05-14

**Authors:** Pablo Ramon Soria, Robert Bevec, Begoña C. Arrue, Aleš Ude, Aníbal Ollero

**Affiliations:** 1 Robotics, Vision and Control Group, University of Seville, Camino de los Descubrimientos, s/n, Seville 41092, Spain; barrue@us.es (B.C.A.); aollero@us.es (A.O.); 2 Humanoid and Cognitive Robotics Lab, Department of Automatics, Biocybernetics and Robotics, Jožef Stefan Institute, Jamova cesta 39, Ljubljana 1000, Slovenia; robert.bevec@ijs.si (R.B.); ales.ude@ijs.si (A.U.)

**Keywords:** UAV, object detection, object recognition, SVM, manipulation

## Abstract

Giving unmanned aerial vehicles (UAVs) the possibility to manipulate objects vastly extends the range of possible applications. This applies to rotary wing UAVs in particular, where their capability of hovering enables a suitable position for in-flight manipulation. Their manipulation skills must be suitable for primarily natural, partially known environments, where UAVs mostly operate. We have developed an on-board object extraction method that calculates information necessary for autonomous grasping of objects, without the need to provide the model of the object’s shape. A local map of the work-zone is generated using depth information, where object candidates are extracted by detecting areas different to our floor model. Their image projections are then evaluated using support vector machine (SVM) classification to recognize specific objects or reject bad candidates. Our method builds a sparse cloud representation of each object and calculates the object’s centroid and the dominant axis. This information is then passed to a grasping module. Our method works under the assumption that objects are static and not clustered, have visual features and the floor shape of the work-zone area is known. We used low cost cameras for creating depth information that cause noisy point clouds, but our method has proved robust enough to process this data and return accurate results.

## 1. Introduction

Unmanned aerial vehicles (UAVs) have been the subject of much research [1] and attracted the interest of the public in recent years. Not only do they offer cost reductions in deployment and operation in a number of scenarios, but they also provide new capabilities in industrial and consumer applications. In order to reduce their dependence on an operator, UAVs have also gained several autonomous capabilities. They are able to autonomously plan paths, cooperate with each other, and even avoid obstacles while flying [2,3,4]. More recently, the interest in developing manipulation capabilities for UAVs has been spurred.

Such a robotic system, also called an aerial manipulator, merges the versatility of multirotor UAVs with the precision of robotic arms. However, the coupling effects between the aerial vehicle and the manipulator gives rise to several modeling and control problems. Several studies have focused on the control of the arm and how it influences the dynamics of UAVs [5,6,7,8,9,10], with the goal of developing a cooperative free-flying robot system for assembly structures. An extension of this idea is under way, which aims to develop the first aerial robots in the world with multiple arms.

Nevertheless, manipulation involves more than the control of an aerial manipulator. In order to grasp and manipulate objects, the robot must first be able to perceive them. The complementary required skill to aid manipulation is therefore object perception using arbitrary sensors. Methods using different types of markers for detecting objects have been developed, e.g., radio markers [11] or visual printed tags [11,12]. In this article, however, we focus on object detection methods that do not rely on additional visual cues but solely on the object’s visual characteristics. Since drones are often used for surveillance tasks, many methods of detecting and tracking objects have been proposed. Some look for motion in images as a cue for object detection [13,14,15,16]. Others use color and intensity information [17,18]. These methods apply for objects that are relatively far away from a high flying drone. We want to solve the task of locating an object in close proximity to the drone, where it can reach it. In order to attempt to grasp an object, the UAV has to acquire some three-dimensional (3D) information about the scene; therefore, the methods mentioned above do not suffice.

Such 3D information is usually gathered in a map of an area. There are several approaches to creating general 3D maps, usually simultaneously localizing the viewer in the scene (SLAM). Some of these approaches use monocular systems [19,20,21], stereo cameras [19,22,23], depth cameras (or RGB-D sensors) [24], or even laser sensors that return very accurate distances to objects in the scene [25]. The task at hand, however, is not to accurately map a large area, but to return the objects pose relative to the drone, so that the drone can use that shape information in order to grasp the object.

There are several methods to describe objects in order to obtain grasping data. For the sake of the greater generality, we are interested in methods that do not require object CAD models (or computer-aided drafting models) to initiate the grasping. Using sparse depth information about an object a Gaussian processes can be used to describe its implicit surfaces [26,27]. The main advantage of this approach is that it provides a guess of the surface of the object and also offers a measurement of uncertainty of the shape, which can be used to decide where to further inspect the object. It has also been shown that when dealing with novel objects, a reactive grasping mechanism can be used to grasp objects using a humanoid robot by determining its dominant axis and centroid [28,29].

**Figure 1:**
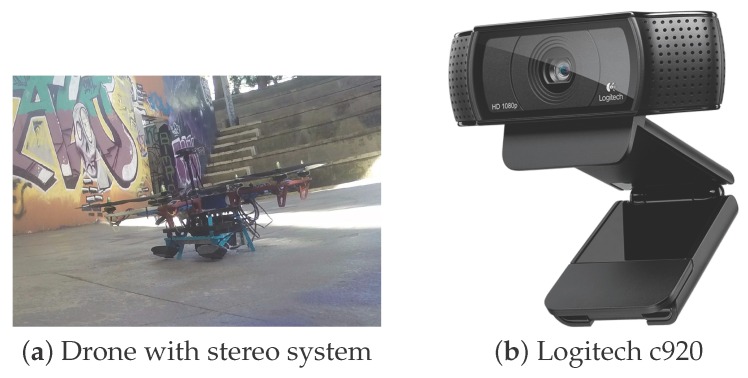
The UAV (**a**) used in the experiments with a stereo system comprised of low cost cameras; (**b**) More information about the system is shown is Section 3.

In this paper, we propose a method that extracts objects from a local map of the work-zone, generated using depth information. Candidate objects are extracted from this map by detecting areas different from our floor model. The candidates are then evaluated using their projections in the color images, where the object classification is executed. Our method builds a sparse cloud representation of each object and calculates the object’s centroid and dominant axis. This information can be passed to a grasping module for a grasping attempt. Our code is suitable for on-board execution and ought to be initiated after the drone has approached the work-zone. The principle of how the drone comes to the actual pick-up location, within two meters of the objects, is beyond the scope of this paper. In our implementation, we use extremely low cost USB cameras, as seen in Figure 1, which capture images using a rolling shutter and do not have control over image triggering and focus. We show that our system is robust enough to successfully tackle the effects produced by these affordable cameras.

## 2. Methodology

### 2.1. System Description

**Figure 2:**
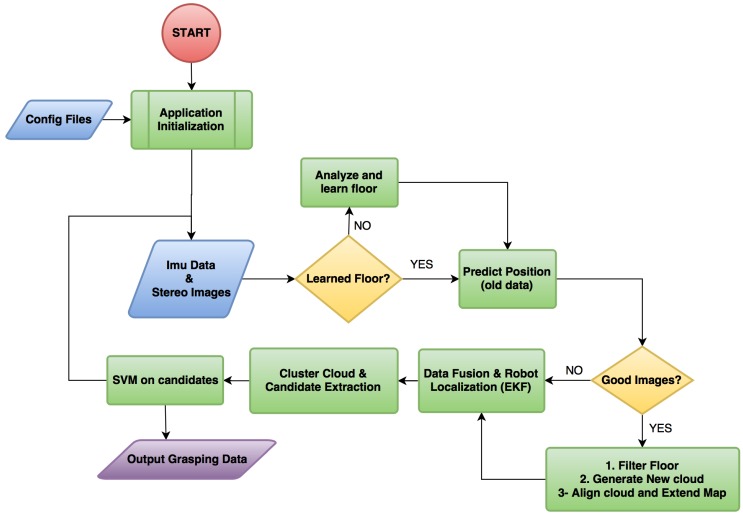
System flow chart.

The general principle of our method is best understood by following the flow chart in Figure 2. The system is initialized once the drone is within two meters above the work-zone floor for our cameras to return usable depth information. Our method requires input from the camera and the inertial measurement unit (IMU) with integrated compass data as explained in Section 2.2. The robot determines whether it is necessary to learn the floor appearance model as described in Section 2.4. The robot then predicts its current position in the map, depending on its previous movement (Section 2.6) and determines whether the images are blurry [30]. Due to motion blur or focus hunting, the images might be useless, and, therefore, the robot does not waste time processing them and goes straight to the Extended Kalman filter module to update the UAV’s current pose (Section 2.6). If the images are in focus, the robot first excludes the floor from the images in case the floor appearance model has been learned (Section 2.4). Afterwards, a point cloud is generated as explained in Section 2.3 and aligned to the map (Section 2.5). The robot then extracts candidate objects from the map and attempts to classify them using a support vector machine (SVM) classifier, as described in Section 2.7 and Section 2.8, respectively. Lastly, at the end of each loop, the object data is returned for a grasping attempt (Section 2.9).

### 2.2. Data Acquisition

Our system requires information from two different sensors:

1. Stereo cameras.
2. Inercial measure unit (IMU) module with compass data (Accelerometer, Gyroscope).

We focused on using cheap stereo cameras that do not have trigger control, which results in unsynchronized stereo images. In the execution loop, the cameras are prompted to return new images and the time difference between them can be anything up to 1/FPS. The drone moves rather slowly above the objects while inspecting them, but larger time shifts are still noticeable and they result in poor point clouds. In Section 2.5, we describe how we deal with noisy clouds, and it is possible to see examples of good and bad clouds in Section 3. The IMU unit provides acceleration and orientation information. The latter can be used directly to estimate the robot’s orientation, while the acceleration data is corrected for gravity and fed into an Extended Kalman filter for the motion model (Section 2.6).

### 2.3. Point Cloud Generation

In order to satisfy a broad spectrum of applications, an affordable depth sensor is required to gather 3D Rdata. Common off-the-shelf depth sensors, such as the Kinect, do not work well at short distances under 1 m [31,32], which makes them difficult to use for manipulation by drones with short arms. An alternative is to use stereoscopy to recover 3D information, while also acquiring 2D color information. Our proposed method works with any method of acquiring depth information and color images; however, we used low cost unsynchronized USB web cameras for the task.

In this implementation, the generation of the point clouds is divided into three steps:

1. Visual feature detection in the left image.
2. Template matching in the right image.
3. Triangulation.

**Figure 3:**
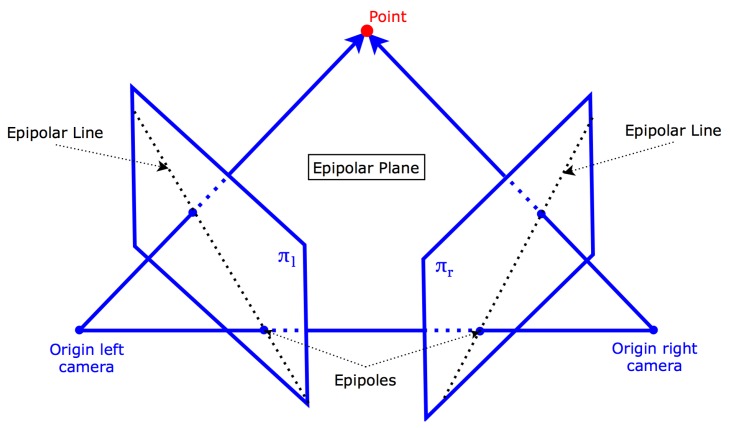
Epipolar geometry.

Keypoints or visual features are distinctive points in an image that are invariant to small changes in view. Keypoints extracted from one stereo image should therefore also be distinctive in the other image. Our camera pair is calibrated, therefore we can use the constraints of epipolar geometry to look for keypoint matches. A template window is slid across the epipolar line and compared to the template of each corresponding keypoint. If the matching score is sufficient, a keypoint pair is then triangulated (Figure 3). All the triangulated keypoints make up the point cloud.

There are several feature detectors to choose from and several metrics for template matching. Often, features like scale invariant feature transform (SIFT) [33] or speeded up robust features (SURF) [34] are used. These features were designed to be robust in order to track them reliably over longer periods of time. However, we require features that are calculated quickly and we need to detect many of them in order to create a more dense point cloud. For this purpose, we chose the Shi–Tomasi corner detector [35] in combination with the squared sum of differences for template matching, but an arbitrary detector can be applied.

### 2.4. Floor Detection and Extraction

Floor extraction is analogous to the background subtraction problem, which is tackled frequently in surveillance tasks. The initialization of the background model is crucial to ensure foreground objects can be extracted effectively. Current state-of-the-art methods such as [36,37,38,39] propose different algorithms for the initialization and maintenance of the background model. However, these approaches assume static cameras. Some methods consider camera movement intrinsically as in [40,41]. Authors in [42] introduce a Bayesian filter framework to estimate the motion and the appearance model of background and foreground. Others like [43] tackle the problem using an optical flow algorithm to segment the foreground objects. All mentioned methods require moving objects in order to extract them from the background, which is not the case in our scenario, where a UAV tries to pick up a static object.

Our method works under the assumption that the floor shape model is known in advance. After the initial 3D map is generated from the point clouds, we use the random sample consensus (RANSAC) algorithm to find the best match of the floor model in the map [44]. Extracting the floor is very important, as it helps to segment the cloud into candidate objects. Only the floor shape model is assumed, but the robot also learns a color and texture model in order to extract it from the images. Floor extraction from the images is done due to the fact that visual features can appear overwhelmingly only on the floor. Our method of point cloud generation relies on visual features appearing on the objects in order to detect them. Distinctive keypoints appear predominantly on the floor, when it has small repetitive patterns, e.g., a gravel floor or a pebble floor. Our method looks for the maximum *N* best features for point cloud generation in order to satisfy the time constraints and the quality of the floor features can completely overwhelm the object features. We consider the following scenarios regarding the floor:

1. The floor in the scene is uniform so it has few features on it.
2. The floor has a texture that can be modeled/learned.
3. The floor has a texture that cannot be learned.

The first scenario is the simplest. The feature detector will mostly find keypoints that correspond to objects and will produce good and accurate 3D points. In this case, RANSAC will not detect a good floor match, but the pipeline will continue to work flawlessly and extract candidate objects.

In the second scenario, the floor has some textures that produce keypoints. RANSAC is able to detect the floor in this case and the robot tries to learn the floor appearance model. Repetitive small patterns in particular cause a lot of problems. However, the good thing is that these patterns can be learned and extracted from the images [45] before creating the 3D cloud. It is important to notice that cropping the floor at this stage will speedup the system as fewer features are detected in the remaining image, so the matching, triangulation and then aligning to the map takes less computational time.

Finally, the third scenario has the same problem with dominant features on the floor as the second. In this case, it is not possible to learn the floor pattern for some reason. This is a less likely event, but if a floor has great variance, it can occur and has to be considered. RANSAC extracts the floor model from the map, but at least some keypoints on the objects have to be found for the system to be able to extract objects. Since geometric parameters of floor are detected, we can exclude the points of the map that belong to the floor and pass the result on to the procedure for candidate extraction. In this scenario, the number of object keypoints will obviously be smaller due to most of them being part of the excluded floor; however, as shown later in Section 3, our system can handle these types of scenarios as well.

**Figure 4:**
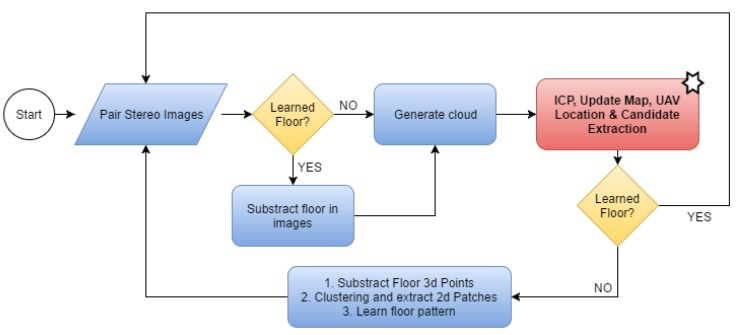
Floor detection and extraction flow chart.

The system automatically detects whether the floor models can be learned in order to extract the floor either from the images (second scenario) or in 3D (third scenario). In all other cases, the system processes the entire map as in the first scenario. Figure 4 gives a the detailed flow chart of our floor learning method with the starred block representing all the other processes in the loop.

### 2.5. Temporal Convolution Voxel Filtering for Map Generation

Point clouds generated by matching features from stereo cameras include noisy points due to bad matches, triangulation and calibration errors, mistimed stereo images (Unsynchronized stereo produces a delay between the captured frames), occasional rolling shutter effect because of vibrations, and even some partially blurry images that get through to this point. For this reason, it is necessary to process these clouds before adding them to the map. We developed a method that processes sequential point clouds in both spatial and time dimensions before adding the result to the map. Due to the movement of the UAV, sequential point clouds first need to be aligned properly using affine transformations. Section 2.6 will explain how this transformation is obtained. After the alignment of the point clouds, we filter out the bad points using our probabilistic map generation procedure based on a sequence of the *N* previous point clouds.

As mentioned, the point clouds are filtered in two steps: (1) Spatial filtering: Isolated particles or small clusters of particles are considered noise (using [46]) and the remaining points are transformed into a grid of cubic volumes of equal size, also called voxels, where a voxel is occupied if at least one point from the point cloud belongs to it [47]; (2) Time filtering: We propose a filtering method over time using sequential voxel point clouds stored into memory, also called history. The occupancy of each voxel is checked in each cloud in history, so that only voxels that have a higher probability of being occupied by a real point will be kept. We call this method Temporal Convolution Voxel Filtering (or TCVF).

Given a set of *N* consecutive point clouds $PC_{i}$, the goal is to obtain a realistic representation of the environment by filtering out incorrect points. The Algorithm 1 describes the process.

**Figure 5:**
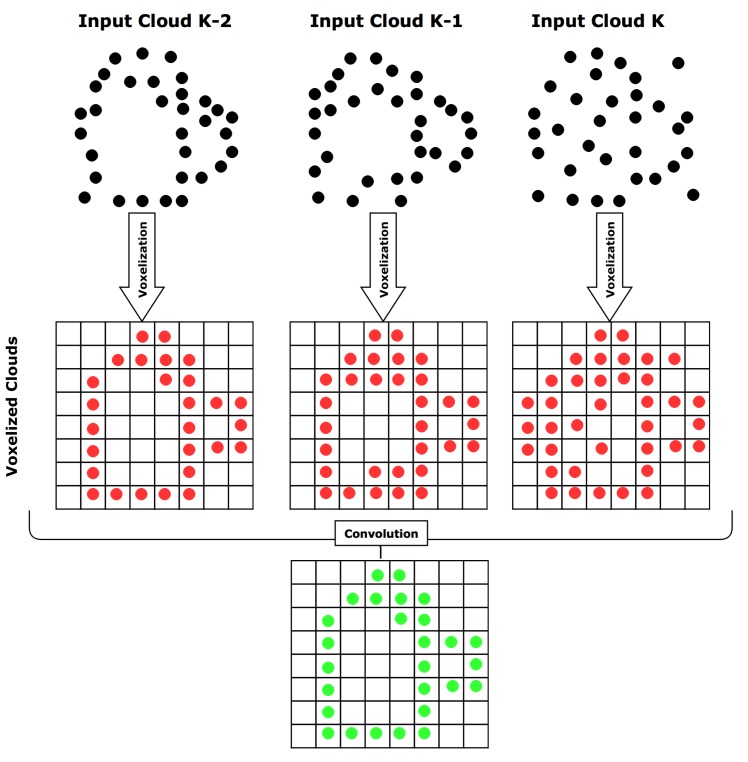
A 2D example of our Temporal Convolution Voxel Filtering for history size of 3. The method checks the occupancy of each voxel through the history of point clouds and only voxels occupied in the entire history are passed through the filter.

TCVF adds a new cloud to the history in each iteration and evaluates the clouds kept in the history at that moment. The result of this operation is then added to the map. By discretizing the space, the number of points for computation is reduced, which reduces the computational time. We use an occupancy requirement of 100% throughout the entire history, making this calculation a simple binary operation of occupancy check, which is very fast and is only evaluated on occupied voxels, making this method computationally light. The number of operations is O(nk), with *n* the number of occupied voxels in the smallest cloud in the history and *k* the history size. The voxel size is predetermined and represents the resolution of our map. Figure 5 shows a schematic of a 2D example using a history size of three.

**Algorithm 1** Probabilistic Map Generation.

MAP←empty**for** i∈[0,N] **do**   $PC_{I}\leftarrow \text{filter}\left(PC_{I}\right)$   $PC_{I}\leftarrow \text{align}\left(PC_{I}\right)$   $PC_{I}\leftarrow \text{voxel}\left(PC_{I}\right)$   $\text{addToHistory}(PC_{I})$**end for****for** $point\;in\;PC_{i}$ **do**   **if** $point\;\exists \;in\;PC_{i}\;with\;i\in \left[0,N\right]$ **then**      MAP **add** point   **end if****end for**


### 2.6. Drone Positioning and Cloud Alignment

At the start of the application, the drone acquires the first point cloud and initializes an empty local map. Since we do not want noise in our map, we use our TCVF algorithm to add points to the map. TCVF needs to first fill the entire history with sequential point clouds in order to determine whether specific points exist. However, the drone is not static, so from the camera’s point of view, the points might move, even though they represent the same actual static point. The camera origin of a point cloud effectively represents the relative position of the drone to the detected scene. Obviously, the sequential clouds must be aligned in order for TCVF to work. The effect of aligning sequential clouds is also an assessment of the updated position of the drone. We use the iterative closest point (ICP) algorithm, which minimizes the distances of pairs of closest points in an iterative fashion, to align point clouds. However, ICP algorithms have difficulties detecting the correct transformation between two sequential point clouds if the change in pose between them is large.

This problem can be solved by using IMU data from the drone to provide an assessment of the pose change and feed this to the ICP algorithm. Unfortunately, it is not possible to rely solely on the IMU data for positioning in GPS-denied environments, because it tends to drift quickly. Luckily, the ICP result gives us an estimation of the drone position, so we implemented an algorithm to fuse the information from the IMU and the ICP result to estimate the position of the drone in the map.

Traditionally, an Extended Kalman Filter (EKF) is used to fuse the visual and inertial data. The result of using an EKF is a smoothed pose estimation. There are several implementations of this idea [48,49,50,51]. In particular, in [51], the effect of the biases in the IMU is studied and a solution provided. Suppose that the system’s state is:

(1) $$X_{k}=\left\{x_{k}^{x},x_{k}^{y},x_{k}^{z},{\dot{x}}_{k}^{z},{\dot{x}}_{k}^{y},{\dot{x}}_{k}^{z},{\ddot{x}}_{k}^{x},{\ddot{x}}_{k}^{y},{\ddot{x}}_{k}^{z},b_{k}^{\ddot{x}},b_{k}^{\ddot{y}},b_{k}^{\ddot{z}}\right\}$$

and the observation’s state:

(2) $$Z_{k}=\left\{x_{k}^{x},x_{k}^{y},x_{k}^{z},{\ddot{x}}_{k}^{x},{\ddot{x}}_{k}^{y},{\ddot{x}}_{k}^{z}\right\}$$

while the equations for the system and the observation are:

(3) $$\left\{\begin{array}{cc} x_{k}^{i}=x_{k-1}^{i}+\Delta t{\dot{x}}_{k-1}^{i}+\frac{\Delta t}{2}{\ddot{x}}_{k-1}^{i}, & i=x,y,z\\ {\dot{x}}_{k}^{i}=\Delta t{\ddot{x}}_{k-1}^{i}, & i=x,y,z\\ {\ddot{x}}_{k}^{i}={\ddot{x}}_{k-1}^{i}, & i=x,y,z\\ bias_{k}^{{\ddot{x}}^{i}}=\frac{T}{T+\Delta t}bias_{k-1}^{{\ddot{x}}^{i}}+\frac{\Delta t+T}{T+\Delta t}\left(C_{1}+C_{2}\right), & i=x,y,z \end{array}\right.$$

(4) $$\left\{\begin{array}{cc} X_{k}^{i}=Z_{k}^{j}, & i=j=0.2\\ X_{k}^{i}=Z_{k}^{j}, & i=0.2,j=3.5 \end{array}\right.$$

Introducing these equations into the EKF allows for predicting the current state of the system. This information is used to locate the cameras in the environment. It is also used to provide a guess in the next iteration of ICP, by taking the current state and assessing the drone’s position after Δt. The orientation is taken directly from the IMU, since it is provided by the compass and does not drift.

Figure 2 shows the pipeline of the whole system and illustrates how the EKF information is used for drone positioning and cloud alignment:

1. The previous state $X_{k-1}$ is used to obtain ${\tilde{X}}_{k}$, which is a rough estimation of the current position of the robot.
2. If the stereo system has captured good images, a point cloud is generated and aligned with the map using ${\tilde{X}}_{k}$ as the initial guess. The transformation result of the alignment is used as the true position of the drone ${\hat{X}}_{k}$. The obtained transformation is compared to the provided guess and discarded, if the difference exceeds a predefined threshold.
3. If the stereo system has not captured good images, it is assumed that ${\tilde{X}}_{k}$ is a good approximation of the state, so ${\hat{X}}_{k}={\tilde{X}}_{k}$
4. The EKF merges the information from the ICP ${\hat{X}}_{k}$, with the information from the IMU, ${\hat{\ddot{X}}}_{k}$, and the resulting $X_{k}$ is the current filtered state.

### 2.7. Candidate Selection

**Figure 6:**
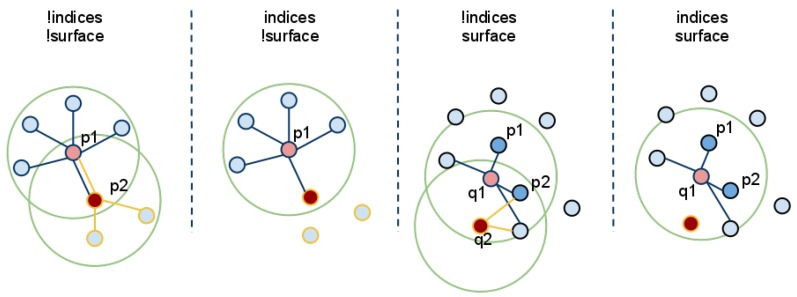
A visualization of the Euclidean cluster extraction [53].

As the robot builds the representation of the environment in the map, the search for candidate objects can be executed. The input cloud for this processing module has already had the floor points removed or has very few floor features as described in Section 2.4. Candidate objects are extracted from the cloud using a clustering algorithm based on Euclidean distances [52]. This clustering method extends each cluster if a point appears closer than a predetermined threshold to any current point in the cluster. An example of this procedure can be seen in Figure 6. Clusters that meet the minimum number of points requirement are selected as candidate objects. The idea behind this is that a tight cluster of features that is not part of the floor could represent an object. Assuming the objects are not cluttered, several candidate objects are extracted and passed to the object recognition module for validation.

### 2.8. Object Recognition

**Figure 7:**
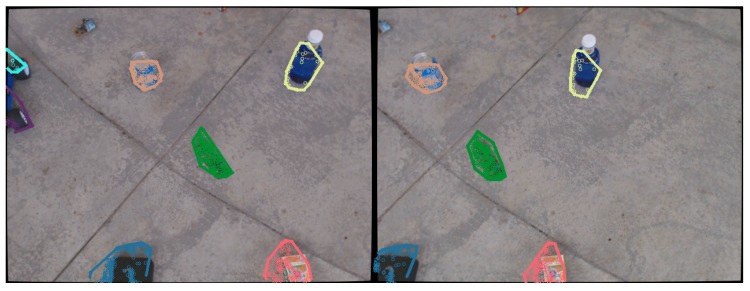
Reprojection of the points belonging to candidate objects, surrounded by a convex hull.

This is the final step of the object detection and recognition system. After the candidate extraction, their point clouds are projected onto the images from the cameras. Due to the accurate cloud alignment and drone positioning system described earlier, the projected points correspond well to their respective objects as seen in Figure 7. For each cluster of 2D points, a convex hull that envelops the object is generated, and a patch is extracted for object classification.

The recognition system is based on the *Bag of Visual Words* (BoW) model [54]. Each object is represented by a histogram of visual descriptors, computed by detecting features in the extracted image patch. We used the Shi–Tomasi corner detector in combination with the SIFT descriptor [33] to describe each object.

The BoW model requires a vocabulary, which is a set of representative descriptors that are used as a reference to quantify features in the images. The vocabulary is generated during the offline classifier training process, where the algorithm detects all features in the training input set and extracts a representative set of *words*. During the training process, an SVM model is trained using positive and negative object samples. The negative samples are used in order to reject false candidate objects, for example if a big enough cluster appears in the map due to noise or a bad floor model. The resulting object detection and recognition system returns the labels of the detected objects, which the drone users can use if a specific object must be picked up or located.

The BoW model has been chosen due to another useful characteristic. It has been shown that it is also good for learning more general representations, like object categories. By training the object to recognize object categories, a novel object can also be classified.

### 2.9. Grasping Data

Each object is represented by a cluster of points. The grasping module can process this information arbitrarily. However, for purposes of illustration and evaluation, we provide the grasping data in a similar form to that defined in [29]. A grasp is planned by calculating the mean position p and the dominant principal axis a of the object. The dominant axis is used for the robot to grasp the object on the narrow side and the centroid is the grasping approach target. Let $x_{i}=\left[x_{i},y_{i},z_{i}\right]$ be the position of *N* cluster points:

(5) $$p=\frac{1}{N}\sum_{i=1}^{N}x_{i}\;$$

By calculating the principal axes of the object’s points, we estimate the object’s greatest extent in each direction. First, the covariance matrix Σ is calculated as:

(6) $$\Sigma =\text{cov}\left(\left\{x_{1},x_{2},\ldots x_{N}\right\}\right)=\frac{1}{N-1}\sum_{i=1}^{N}\left(x_{i}-p\right){\left(x_{i}-p\right)}^{T}$$

Next, we calculate the three eigenvalues $\lambda_{1},\lambda_{2},\lambda_{3}$ and eigenvectors $v_{1},v_{2},v_{3}$ of the covariance matrix Σ, which is done by solving the equation

(7) $$\left(\Sigma -\lambda I\right)v=0$$

The eigenvector associated with the biggest eigenvalue represents the dominant axis a of the object.

## 3. Experimental Validation

We performed several experiments to evaluate our proposed method. A pair of Logitech c920 cameras [55] were mounted on the bottom of a hexacopter as seen in Figure 1. The drone is equipped with a Pixhawk IMU [56] for the inertial measurements. The baseline of the cameras was approximately 20 cm and they were facing towards the floor at an angle of 70 to the horizon. The detailed specifications and parameters of the system can be found Appendix A. A human operator controlled the drone during flights and the initialization of our method was triggered manually. An Intel NUC [57] computer with a 5th generation i7 processor was on board for vision processing, and our method was able to run at about five frames per second.

The experiments were executed in four different scenarios, summarized in Table 1. In all examples, our floor model was a plane. The *Laboratory* scenario was executed on a white uniform floor with very few visual features, where the drone was hand-held and moved manually. Due to the white floor, this scenario does not trigger our floor extraction module, and there is no vibration from the motors to cause image acquisition problems.

The *Street 1* scenario was again hand-held, but executed outside on a gravel floor. This floor was full of visual features, an example where features are detected predominantly on the floor as described in Section 2.4. The floor extraction module detected the floor within the first three iterations and learned its appearance, removing it from the images before creating point clouds.

**Figure 8:**
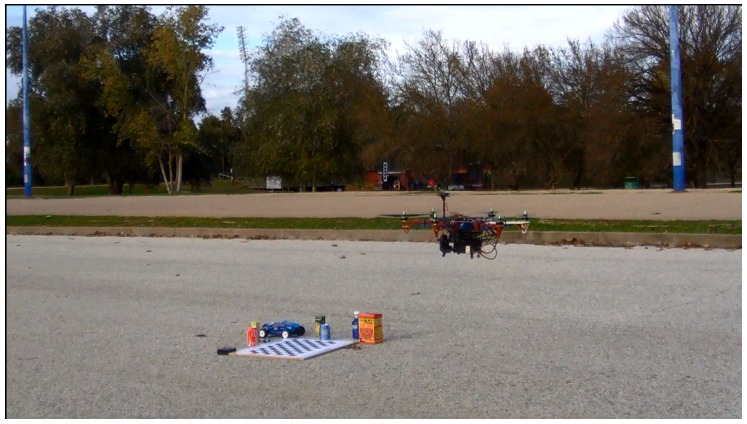
The drone flying above the objects during an outdoor experiment.

In the *Street 2* scenario (Figure 8), the drone was airborne, flown by an operator above a floor with similar texture as in *Street 1*. This scenario represents an example, where vibrations are generated by the motors and images may suffer from the rolling shutter effect. The effect results in bad point clouds.

Lastly, the scenario *Testbed* was executed in an indoor test facility for drones with a VICON [58] measuring system. The drone flew above a complex textured floor, where the ground truth of all of the objects’ positions and the drone motion was measured by VICON. This represents the most difficult scenario for our method, where the floor has many visual features, but it is not possible to learn its appearance and the motors are running and causing vibrations. Our method has to deal with bad point clouds, where few visual features belong to actual objects.

Table 2 summarizes the results of our object extraction method after 15 s of flight. In our scenarios, the number of total points in the local map stopped, increasing significantly after this point, meaning the area had been mostly inspected and the results have converged. However, this is an empirically derived value that depends on the diversity of the observed scene and the flight path the UAV takes to inspect it.

**Figure 9:**
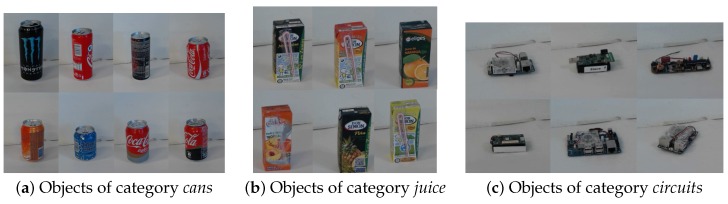
Our test objects were also grouped into categories. We see category examples belonging to *cans* in (**a**); *juice boxes* in (**b**) and *circuits* in (**c**).

The SVM classifier was trained using 16 individual objects, and, in the other case, the objects were grouped by categories (Figure 9): cans; juice boxes; circuits; cars; boxes; Some of the objects were very similar and hard to distinguish from certain angles, e.g., the original coke and generic copy, juice boxes of the same brand but different flavors, since they possess intentionally similar appearance. In the *Laboratory* scenario with a plain background, the results for individual object recognition were good and perfect when the objects were grouped in categories. It should be noted that we used no color information for training and recognition, although it could improve recognition results of individual objects. In the other scenarios, the recognition rates were lower, and we attribute this to our feature extraction implementation. We used a square bounding box around the objects to extract features instead of only the convex hull. In training, the background was plain, but for those examples with a textured floor, a lot of the background is included in the bounding box, and it is very rich with visual features. Still, we can see that the categories were detected better than the individual objects.

Because our system works with low-cost unsynchronized USB cameras with auto-focus, some pairs of images are not useful. Blurring can occur due to motion or focus hunting, the timing difference between the pair can be too big and occasionally even distortions appear due to the vibrations producing the rolling shutter effect. There are two possible outcomes. If our blur detection module registers a blurry image, we omit it and get a new pair. However, in low light, when the shutter speed of the camera is decreased, motion blur can be present for a longer time. This poses a problem for the ICP algorithm, which requires a good guess for successful alignment. If the position of the drone is lost, the guess cannot be provided and the ICP fails. Our positioning system ensures that the position of the drone is estimated in the EKF using IMU data. The ICP is then able to align a new cloud after a longer period of blurry images and recover the drone’s true position. Due to drift, the IMU based guess is not perfect, but, without using it, recovery is unlikely.

**Figure 10:**
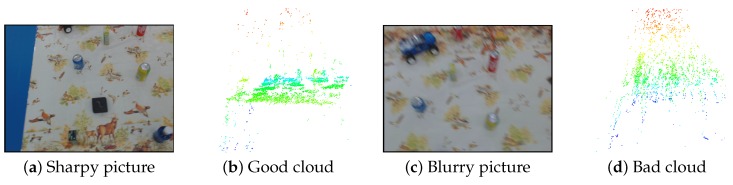
(**a**) shows a good input image. A point cloud generated from good images is shown in (**b**); where the floor plane is clear with objects protruding out; (**c**) shows a blurry image that generates a poor quality cloud (**d**); where the floor and objects are indistinguishable. A similarly bad cloud is also generated when images are mistimed too much.

The other possible outcome is that the drone generates a point cloud from the bad images. In that case, the quality of the cloud decreases drastically as shown in Figure 10. However, the TCVF is able to handle such clouds and does not compromise the map with noisy data. A noisy point would have to appear in the same voxel throughout the entire history of *k* for it to appear in the map. In our experiments, we used empirically obtained parameters of history k=3 and voxel size 5 mm.

**Figure 11:**
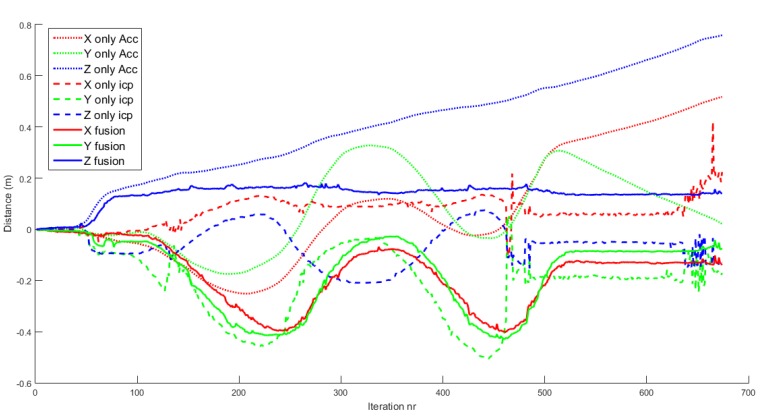
Comparison of drone positioning using the EKF with: only IMU data; only ICP results; fused IMU data and ICP results. Using only IMU data, the position drifts away quickly. Using only ICP results, the position has several bad discrete jumps and does not correspond to actual motion. Using the fused data, the position corresponds to actual motion.

The positioning system of the drone relative to the objects is one of the key issues. Figure 11 shows how the fusion of ICP information and IMU information gives more robust and accurate results than using only IMU data or ICP results separately. The RGB dotted lines refer to the XYZ estimation of position using only IMU information. As mentioned in Section 2.6, this tends to drift due to the accumulation of errors. The RGB dashed lines are the XYZ positions using only ICP. These results are initially good in so far as all the input clouds are confident. In iteration 120, the algorithm converges to a wrong solution, and then it does not recover. The RGB solid lines are the XYZ positions of the drone for the fusion algorithm. It returns stable and robust estimates of the position of the drone.

**Figure 12:**
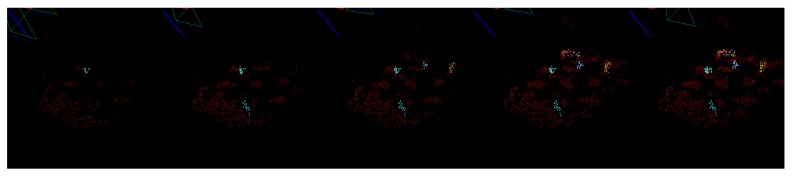
The local map in different iterations. The size of the map is increasing (**red** points) and new object candidates are discovered (seen in color).

**Figure 13:**
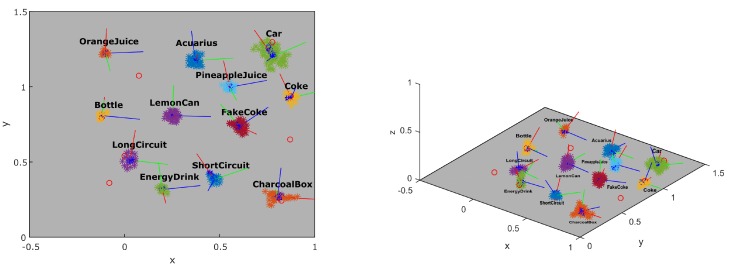
Reconstruction of the objects in the scene (**colored** clusters) compared to ground truth (**red** circles). The frame in each object represents the PCA (principal component analysis) results with the red axis representing the dominant axis.

The progress of building the local map of the scene can be seen in Figure 12. The number of features in the map grows and candidate objects appear defined by points of the same color. In order to analyze the quality of the object localization, we compared the results to the ground truth acquired using the dataset *Testbed*. Figure 13 shows the resulting position of the candidates in the scene after approximately 15 s of inspection. Each colored cluster represents a candidate with a PCA defined coordinate system of its pose in the center. The red circles represent the ground truth position of the objects. Three objects were not detected in this dataset.

**Figure 14:**
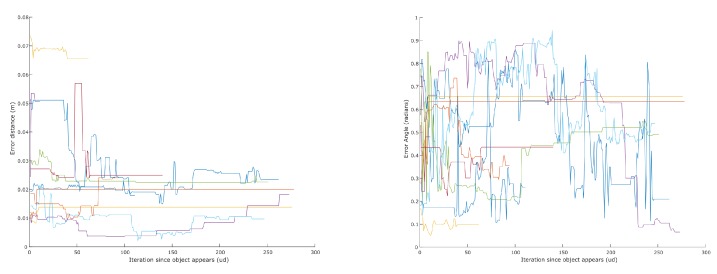
Error of extracted objects’ centroid (**left**) and orientation (**right**). The moment of object discovery is aligned to zero on the abscissa. If an object disappears from the view, its plot ends but its last pose is kept.

**Figure 15:**
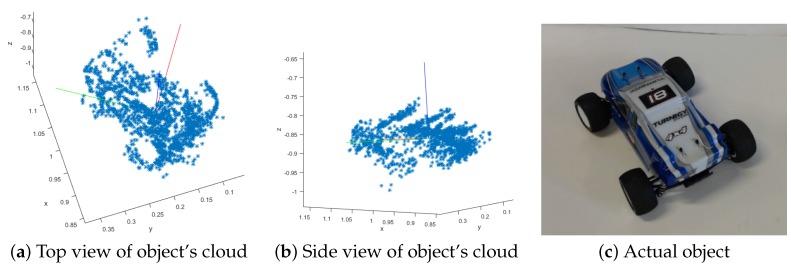
Example of an extracted object. (**a,b**) show the point cloud from different angles; (**c**) is a picture of the actual object.

In order to evaluate the grasping information returned by our system, we calculated the error between each candidate and the ground truth in position and orientation. Figure 14 shows how these errors evolve over time for each object. The position error decreases or remains very stable. When a candidate is first discovered, it has fewer features and might not contain features seen from different angles, rendering its centroid less accurate. As the object is inspected from different angles, this centroid improves accordingly. Similar behavior is noticeable with angles. When a complete representation of the object is acquired, the accuracy of the centroid and orientation is very good. Table 3 shows the average error and variance of the object centroids and orientation after observing the scene for about 50 s. An example of an extracted object can be seen in Figure 15.

## 4. Conclusions

We have developed an on-board object extraction method for rotary wing UAVs that calculates the information necessary for autonomous grasping of objects, without the need of providing a model of the object’s shape. An SVM classification procedure is used to recognize specific objects or reject bad candidate objects in a work-zone populated with several objects. Our method works under the assumption that the objects are static and not clustered, have visual features, and that floor shape model of the work-zone area is known. The low cost cameras we have used for creating the depth information occasionally cause very noisy point clouds, but our method for creating a local map has proved robust enough to process this data and return accurate results.

There are several applications for our method to work under the previously mentioned assumptions, particularly industrial applications in partially unknown environments. A drop-off/pick-up zone for arbitrary objects can be selected and the drone ought to pick up objects autonomously without requiring any information about the exact location and shape of the object in advance. A limitation of our method is the time the UAV can handle without accurate point clouds due to either blurry images, the rolling shutter effect or unsynchronized stereo. Blurry images are not used for point cloud generation, while point clouds produced from mistimed or distorted images result in incorrect ICP alignment results. The UAV discards bad ICP results as described in Section 2.6 and must rely solely on the IMU data, causing a slow drift in position estimation. Eventually this drift is so large that the position cannot be recovered anymore due to ICP failure. We can tackle this limitation by improving the camera sensors, reducing the number of bad images, which improves the stability of the system for observing the scene.

A particular advantage of our system is that we extract objects using a bottom-up approach, where candidate objects are extracted from a stereo reconstructed local map of the scene and then recognized using 2D information from the images. Our method is therefore also applicable to the localization and grasping of completely novel objects, since no knowledge about the object is needed for the generation of the candidate objects. The manipulation module can then work in unison with our system to validate candidate objects with information acquired during grasping.

A lightweight arm must be designed and mounted on our UAV in order to test the grasping module that uses the information returned using our proposed method. We plan to design a control method for flying the UAV over objects in a way that will optimize building the local object map and the recognition of the objects. Currently, an operator either flies the UAV manually or predetermines a way-point flight plan. Ultimately, we want to enhance our method to handle mobile objects too, which represents a significant challenge.

## Figures and Tables

**Table 1:** Description of the testing scenarios.

Scenario	Location	Movement	Floor Type
Laboratory	indoor	hand-held	white uniform
Street 1	outdoor	hand-held	gray textured
Street 2	outdoor	flight	gray textured
Testbed	indoor	flight	textured complex

**Table 2:** Results of the object extraction method.

	Categories			Objects		
**Dataset**	**Precision**	**Recall**	**F-Score**	**Precision**	**Recall**	**F-Score**
Laboratory	1	1	1	1	0.5	0.667
Street 1	0.429	0.6	0.6	0.333	0.4	0.36
Street 2	0.783	0.4	0.53	0.75	0.33	0.462
Testbed	0.429	0.5	0.462	0.2	0.167	0.182

**Table 3:** Centroid and orientation error of the extracted objects.

Error	Mean	*σ*
centroid (m)	0.0256	0.1356
angle (rad)	0.3831	0.4320

## Appendix A. Parameters of the System

**Parameter**	**Description**	**Value**
Camera Parameters		
ROI	Usable part of images due to distortion	[∼30,∼30,∼610,∼450]
Blur Threshold	Max. value of blurriness (Section 2.2)	1.1–1.5
Disparity Range	Min-max distances between pair of pixels. This value determine the min-max distances	[∼50,∼300] px → [∼0.3,∼2] m
Template Square Size	Size of the template for template matching	7–15
Max Template Score	Max. allowed score of template matching	0.004–0.05
Map Generation Parameters		
Voxel Size	Size of voxel in 3D space grid	0.005–0.03
Outlier Mean K	Outlier removal parameter	10–20
Outier Std Dev	Outlier removal parameter	0.05–0.1
ICP max epsilon	Max. allowed error in transformation between iterations in ICP	0.001
ICP max iterations	Number of iterations of ICP	10–50
ICP max corresp. dist	Max. initial distance for correspondences	0.1–0.2
ICP max fitting Score	Max. score to reject ICP result	0.05–0.1
Max allowed Translation	Max. translation to reject ICP result	0.05–0.15
Max allowed rotation	Max rotation to reject ICP result	10–20
History Size	History size of TCVF	2–4
Cluster Affil. Max. Dist.	Minimal distance between objects	0.035
EKF Parameters		
Acc Bias Calibration	Bias data from IMU X,Y,Z	−0.14, 0.051, 6.935
Acc Frequency	Mean frequency of data	1 KHz
Gyro Noise	Average magnitude of noise	0.05rad/s
Gyro Frequency	Mean frequency of data	30 KHz
Imu to cam Calibration	Transformation between camera and IMU	*Data from Calib*
Q System cov. Mat.	Covariance of System state variables	*Data from Calib*
R Observation cov. Mat.	Covariance of Data	*Data from Calib*
Recognition System		
Training params	Train parameters of SVM	—-
Detector Descriptor	Feature detector and descriptor used	*SIFT*
